# Progress in small-angle scattering from biological solutions at high-brilliance synchrotrons

**DOI:** 10.1107/S2052252517008740

**Published:** 2017-08-08

**Authors:** Anne T. Tuukkanen, Alessandro Spilotros, Dmitri I. Svergun

**Affiliations:** a European Molecular Biology Laboratory, EMBL Hamburg c/o DESY, Notkestrasse 85, 22607 Hamburg, Germany

**Keywords:** small-angle X-ray scattering, structural modelling, time-resolved SAXS

## Abstract

Small-angle X-ray scattering (SAXS) is an established technique that provides low-resolution structural information on macromolecular solutions. Recent decades have witnessed significant progress in both experimental facilities and in novel data-analysis approaches, making SAXS a mainstream method for structural biology. The technique is routinely applied to directly reconstruct low-resolution shapes of proteins and to generate atomistic models of macromolecular assemblies using hybrid approaches. Very importantly, SAXS is capable of yielding structural information on systems with size and conformational polydispersity, including highly flexible objects. In addition, utilizing high-flux synchrotron facilities, time-resolved SAXS allows analysis of kinetic processes over time ranges from microseconds to hours. Dedicated bioSAXS beamlines now offer fully automated data-collection and analysis pipelines, where analysis and modelling is conducted on the fly. This enables SAXS to be employed as a high-throughput method to rapidly screen various sample conditions and additives. The growing SAXS user community is supported by developments in data and model archiving and quality criteria. This review illustrates the latest developments in SAXS, in particular highlighting time-resolved applications aimed at flexible and evolving systems.

## Abstract 2

Small-angle X-ray scattering (SAXS) is an established technique that provides low-resolution structural information on macromolecular solutions. This review illustrates the latest progress in the field, highlighting analytical methods for flexible and evolving systems, progress in time-resolved SAXS and developments in archiving and validation.

## 1. Introduction   

**Figure 1:**
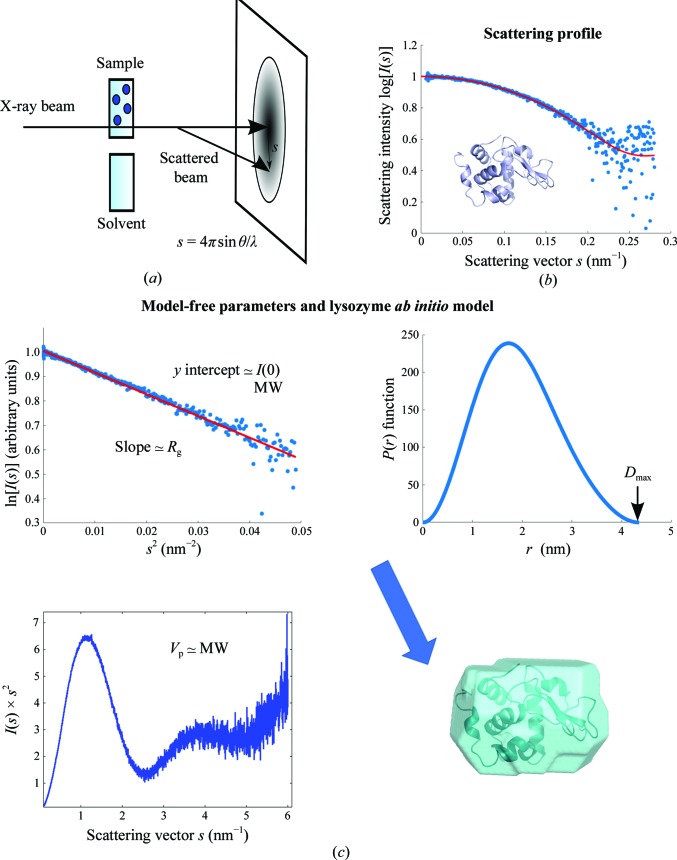
(*a*) Small-angle X-ray scattering (SAXS) experiment. The macromolecular solution is exposed to a collimated, monochromatic beam of X-rays and the angular dependence of the scattered radiation is measured. (*b*) A scattering profile of lysozyme. The lysozyme data (at 6.08 mg ml^−1^ concentration) were collected with an MLM on the P12 beamline at PETRA III using an EIGER 4M pixel detector (frame rate 750 Hz, exposure time 1.3 ms). (*c*) The Guinier plot, *p*(*r*) function, Kratky plot and *ab initio* model of lysozyme. The best known model free parameter is the radius of gyration *R*_g_, which is evaluated from the lowest angles using the classical Guinier approximation *I*(*s*) ≃ *I*(0)exp[−(*sR*_g_)^2^/3] and the linear plot ln[*I*(*s*)] *versus* *s* ^2^ (Guinier, 1939 ▸). *R*_g_ is sensitive to the overall size and shape of a particle and the zero angle scattering *I*(0) (also obtained from the Guinier plot) is related to its MW. The electron pair distance distribution function *p*(*r*) of a molecule is computed using an indirect Fourier transformation of scattering data and yields the maximum size *D*_max_ of a particle (Glatter, 1977 ▸; Svergun, 1992 ▸). Integrating the scattering data and calculating the so-called Porod invariant provides an estimate of the particle volume *V*_p_ (Porod, 1982 ▸). A qualitative indicator of particle flexibility can be obtained by using the Kratky representation where *s* ^2^ *I*(*s*) is plotted against s*s*. Its intensity normalized version, where the momentum transfer is multiplied by the *R*_g_ of a particle, facilitates flexibility comparison between different proteins (Kratky & Porod, 1949 ▸; Durand *et al.*, 2010 ▸). The single-shot exposure lysozyme data can be utilized for standard SAXS analyses including *ab initio* modelling. The fit of the theoretical scattering based on a lysozyme X-ray crystallographic structure (in red, PDB id: 1lys; Harata, 1994 ▸) yields a goodness of the fit χ^2^) = 1.1.

Small-angle scattering of X-rays (SAXS) is a versatile structural analysis method to study biological macromolecules and their complexes and to structurally characterize kinetic processes (Svergun *et al.*, 2013 ▸). SAXS directly provides a number of overall solute parameters and allows structural modelling of the three-dimensional structure of a solute particle based on its measured one-dimensional scattering profile (Fig. 1 ▸). It is applicable to a wide range of molecular weights (MW) starting from a few kilodaltons and extending into the gigadalton range, allowing one to cover objects from peptides to large macromolecular assemblies. In particular, SAXS is able to comprehensively characterize dynamic processes in systems where macromolecular structures are evolving under varying experimental conditions (for example time, pH, pressure or additives; Giehm *et al.*, 2011 ▸; Ryan *et al.*, 2016 ▸; Cordeiro *et al.*, 2016 ▸; Mojumdar *et al.*, 2017 ▸).

In general, a highly purified monodisperse ideal sample without intermolecular interactions is an essential requirement for reconstruction of three-dimensional models from SAXS data. However, no additional labelling, crystallization, freezing or chemical modification of the sample is needed, which makes SAXS universally applicable. The technique is particularly suitable for studies of flexible and disordered proteins, large biomolecular assemblies and oligomeric mixtures, and of the kinetics of processes, which are difficult to investigate using other structural biology methods. Depending on the problem being addressed and the experimental setup used, the amount of purified material required ranges from hundreds of micrograms to a few milligrams. In a typical scattering experiment, the macromolecular solution is exposed to a monochromatic beam of X-rays (or neutrons) and the scattered radiation is detected as a function of momentum transfer *s* = 4πsinθ/λ, where 2θ is the scattering angle and λ is the radiation wavelength (Fig. 1 ▸ *a*). By subtracting the separately measured solvent scattering from that of the solute, the pure signal from the macromolecules is obtained. In dilute solutions, particles are tumbling freely; hence the scattering profile is continuous and isotropic as a result of spherical averaging. A concentration series of samples (typically from about 0.1–0.5 to 5–10 mg ml^−1^ depending on the molecular weight of the particle and on its solubility) is measured in order to account for possible concentration-dependent repulsive or attractive intermolecular interactions.

The most advanced SAXS studies are conducted with synchrotron radiation (SR), and many SR facilities run beamlines that are capable of bioSAXS measurements (for example, ID-18 BioSAXS at APS, USA, BL4.2 at SSRL, USA, SWING at SOLEIL, France, BM29 at ESRF, France, P12 at EMBL/PETRA III, Germany, SR13 ID01 at the Australian Synchrotron, Australia, BL23A at NSRRC, Taiwan, B21 at Diamond Light Source, UK and BL19B2 at SPring-8, Japan). Modern facilities offer high-brilliance and low-background setups for fast (sub-second range) data collection, and recently also robotic sample changers and automated data-analysis pipelines (see, for example, Franke *et al.*, 2012 ▸; Perkins *et al.*, 2016 ▸). The development of single-photon-counting pixel detectors (PILATUS; Kraft *et al.*, 2009 ▸) and next-generation pixel detectors (EIGER; Johnson *et al.*, 2012 ▸) have contributed to the growing use of SAXS in biology. Small pixel sizes, high frame rates and very short readout times provide better accuracy and higher time resolution in data collection. Furthermore, the new detectors and high photon fluxes at SR sources lead to improved signal-to-noise ratios (SNRs) for data, enabling studies of transient complexes present at low concentrations and also accurate measurements at higher angles (*s*_max_ > 5.0 nm^−1^; wide-angle X-ray scattering; WAXS). Additionally, short data-acquisition times permit one to follow the kinetics of biological processes on the microsecond timescale. Besides SR sources, stations based on laboratory X-ray sources are used for biological SAXS (for example, the BioSAXS 2000 instrument from Rigaku or the NANOSTAR instrument from Bruker). They provide lower flux than synchrotrons and are more suitable for static experiments, where the time resolution is less critical. Further advances in laboratory sources are expected to emerge with the broader use of the high-brilliance metalJet X-ray tube technology by Excillum (Hemberg *et al.*, 2003 ▸). Most of the advantages of SAXS also hold for neutron scattering (SANS), which is conducted on neutron reactors or spallation sources. Although neutron experiments typically require more material, valuable additional information can be obtained using SANS thanks to H/D exchange (Whitten & Trewhella, 2009 ▸).

The price to pay for conducting SAXS on near-native solutions is the loss of information owing to spherical averaging, leading to possible ambiguity and low resolution of the models built with SAXS only. Still, many problems in SAXS data interpretation can be overcome by hybrid modelling, integrating data from different sources. The popularity of SAXS-based hybrid approaches coupled with automation of data collection and new methods of analysis has tremendously increased the applications of SAXS in structural biology. This is true in particular for structural characterization of flexible systems and kinetic processes, where SAXS often provides unique information about the system. Here, the major recent advances in the applications of SAXS using SR will be reviewed together with emerging data- and model-validation approaches.

## 2. Structural modelling against SAXS data   

**Figure 2:**
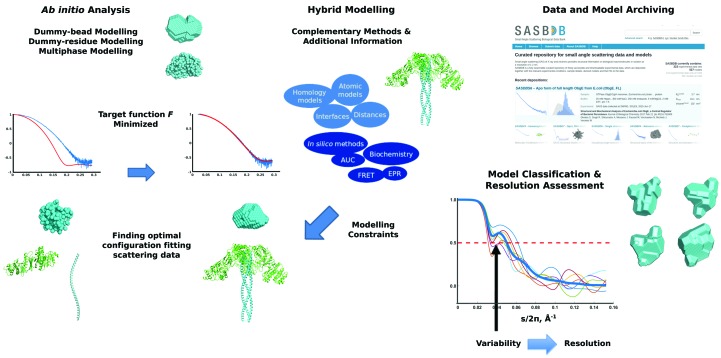
SAXS-based modelling of monodisperse systems. *Ab initio* modelling approaches provide either dummy-bead or dummy-residue models based solely on SAXS data. In case atomic structures (obtained by homology modelling, NMR, EM or MX) of the subunits of a macromolecular assembly or domains of a multi-domain protein are available, hybrid rigid-body modelling can be employed. A target function F containing contributions from the goodness of the fit and available constraints (equations 1 and 2) is minimized by finding an optimal configuration of volume elements, subunits or domains fitting experimental scattering data. SAXS data and models together with quality measures such as their resolution should be freely available to the scientific community and deposited in databases.

Several important overall parameters related to the shape and size of a particle (for example its radius of gyration *R*_g_, maximum size *D*_max_ and volume *V*_p_) can be directly derived from SAXS data (Svergun *et al.*, 2013 ▸; Fig. 1 ▸). However, far more informative are the methods that allow one to reconstruct three-dimensional structural models from scattering data. Structural modelling against SAXS data is an ill-posed inverse problem which can be formulated as a search for an optimal configuration of volume elements (for example beads) or structural fragments (domains or subunits). The model is built such that its theoretical scattering *I*_theor_(*s*) fits the measured scattering of the system *I*_exp_(*s*) to minimize the discrepancy χ^2^, 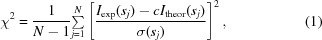
where *c* is a scaling factor, *N* is the number of points and σ denotes experimental error. Acceptable χ^2^ values are around 1.0 provided that the experimental errors are correctly assigned. In general, the reconstruction of a three-dimensional model based on the one-dimensional scattering profile is ambiguous and multiple models may provide equally good fits. The target function *F* to be minimized in the modelling typically also contains a set of penalty functions *P_i_*, demanding additional restraints, 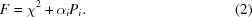
The penalty terms *P_i_* with weights α_*i*_ > 0 enforce physical constraints (for example, interconnectivity and the absence of steric clashes) or take into account additional structural information (for example, contacts between specific residues) in the case of hybrid modelling (Fig. 2 ▸).

χ^2^ statistics are a classical way of presenting the goodness of fit of the model, but this statistic needs accurate standard deviations of the experimental data. If the latter are not available or are incorrectly assigned, the use of χ^2^ may lead to overfitting or underfitting. Recently, an alternative correlation map (CM) approach has been proposed (Franke *et al.*, 2015 ▸), that allows assessment of the goodness of fit without the need for the standard deviations. The CM approach is based on a parametric distribution for the probability of having the longest stretch with a constant sign of the deviation, and provides a probability (*p*-value) indicating the statistical significance of the fit. It was demonstrated that the statistical power of CM to detect systematic deviations is equivalent to that of χ^2^ statistics, even though CM only uses the experimental data and not their associated errors.

### 2.1. *Ab initio* modelling   

In the absence of *a priori* structural data on a system, the one-dimensional SAXS profile alone is able to provide shape information *ab initio*. The first *ab initio* approaches employed simple geometrical bodies or angular envelope functions to describe particle shapes (Svergun *et al.*, 1996 ▸). More versatile shape-reconstruction algorithms utilize models represented by finite-volume elements (Chacón *et al.*, 1998 ▸; Svergun, 1999 ▸). Here, the shape is parameterized by a large number (up to thousands) of beads (Svergun, 1999 ▸; Franke & Svergun, 2009 ▸) or dummy residues (DRs; Svergun *et al.*, 2001 ▸). Starting from a random configuration, simulated annealing (SA) is employed to minimize the target function *F*, yielding a compact shape the scattering of which agrees with the experimental data (equation 2; Fig. 2 ▸). The possibility of imposing constraints *P_i_* (for example symmetry for oligomeric structures) and fitting multiple curves for inhomogeneous models consisting of distinct components, for example protein–nucleic acid complexes, further improve the accuracy of the models (Svergun & Nierhaus, 2000 ▸). At present, *ab initio* modelling has become a rapid and routine procedure that can be performed automatically at SAXS beamlines, essentially inline with the measurements, and can provide structural information immediately after the experiments (Franke *et al.*, 2012 ▸). Most biological SAXS articles now report *ab initio* models; in a recent example the low-resolution shapes of the calcium-binding RTX domain of adenylate cyclase toxin–haemolysin (CyaA) reconstructed solely from SAXS data complemented very successfully other structural biology methods (Bumba *et al.*, 2016 ▸). Here, the combination of circular-dichroism data, the crystallographic structure of a single RTX-repeat β-roll subdomain and the SAXS model of the entire CyaA RTX domain provided mechanistic insights into a ratchet mechanism of protein translocation through the ‘channel-tunnel’ ducts of type I secretion systems.

### 2.2. Hybrid modelling   

If predicted models or high-resolution structures of particles or their domains/subunits are available, SAXS offers broad possibilities for hybrid modelling. Validation of atomic models against SAXS data is one of the typical tasks. *CRYSOL*, the most commonly used program to compute theoretical scattering profiles of structures and compare them with experimental SAXS data, employs spherical harmonics and surrounds the molecule with a continuous envelope of constant adjustable electron density (Svergun *et al.*, 1995 ▸). In later approaches, such as *FoXS* (Schneidman-Duhovny *et al.*, 2010 ▸, 2016 ▸), hydration is represented implicitly based on the fractions of solvent-accessible surface of atoms or by introducing explicit water molecules using MD simulations, as implemented in *AXES* (Grishaev *et al.*, 2010 ▸), *HyPred* (Virtanen *et al.*, 2011 ▸) and *WAXSiS* (Knight & Hub, 2015 ▸). A further important aspect of theoretical scattering computation is the description of the excluded volume that a molecule occupies. In *CRYSOL*, the average displaced volume per atomic group is based on its van der Waals radius and used as one of the fitting parameters. An accurate treatment of the hydration-layer and excluded-volume contributions are especially important when simulating scattering profiles at wider angles (WAXS range; *s*_max_ > 0.5 Å^−1^; Knight & Hub, 2015 ▸) and decomposing the scattering of multi-species systems (see below for more details of flexible and dynamic systems).

Rigid-body modelling based on SAXS data employs available or predicted structural models of individual subunits or domains of a studied system. In addition, information from complementary biophysical techniques (such as light scattering or analytical ultracentrifugation) or *in silico* approaches (for example molecular-dynamics simulations) can be used in hybrid modelling as restraints in (2). The program *SASREF* and its extended version *CORAL* (Petoukhov & Svergun, 2005 ▸; Petoukhov *et al.*, 2012 ▸) allow one to build rigid-body models of molecular assemblies from their components and, if necessary, to add missing fragments (for example N- and C-termini or linkers) using SAXS data. Rigid-body modelling has been extensively employed to provide insights into the structures of protein–protein, protein–RNA/DNA and protein–ligand complexes (Gully *et al.*, 2015 ▸; Cai *et al.*, 2016 ▸; Gógl *et al.*, 2016 ▸). For example, Lerche *et al.* (2016 ▸) obtained models of LysR-type transcriptional regulators (LTTRs), which modify basic metabolic pathways or virulence-gene expression in prokaryotes in complex with promoter–operator region DNA. The SAXS-based models provided new evidence for the ‘sliding dimer’ hypothesis concerning LTTR activation mechanisms. In another study, Hagelueken *et al.* (2015 ▸) examined the mechanism of WbdD from *Escherichia coli*, a membrane-associated protein complex working as a molecular ruler regulating chain lengths in lipopolysaccharide polymerization, using a combination of macromolecular crystallography (MX), *in silico* modelling and SAXS. Sequence analysis revealed a long coiled-coil domain in WbdD, but MX could only resolve the structure of the N-terminal domain with the initial part of the coiled-coil region of the trimeric complex. Using the average coiled-coil radius and the rise per residue obtained from the partial MX structure, a model of the entire coiled-coil domain was built. This model was further employed in a hybrid approach utilizing the SAXS data to produce a structural model of full-length WdbD. The SAXS model together with experiments on protein constructs of the coiled-coil domain of different lengths provided insights into the underlying mechanism of controlling lipid chain lengths that was previously poorly understood. Other relevant examples can be found in recent reviews (Kirby & Cowieson, 2014 ▸; Tuukkanen & Svergun, 2014 ▸; Chen & Pollack, 2016 ▸).

### 2.3. Mixtures and flexibility   

**Figure 3:**
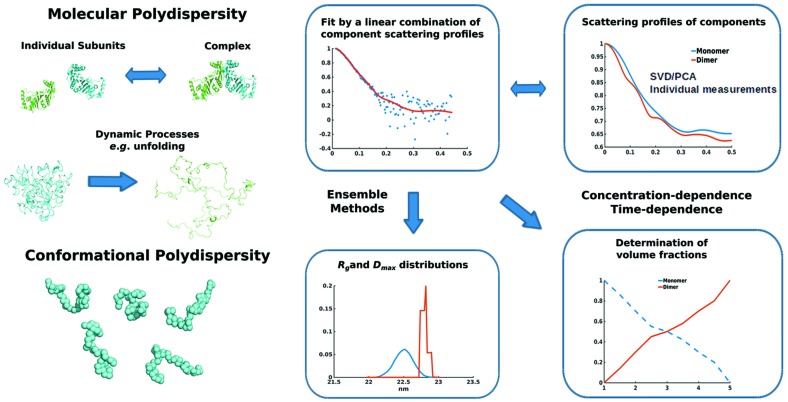
Approaches to study polydisperse systems using SAXS. The polydispersity problem of complex dynamic systems can be solved either by using advanced computational methods or experimental approaches. Scattering profiles of mixtures are linear combinations of component scattering contributions. SVD or PCA decomposition of a measured scattering profile can provide a scattering basis set. In case the scattering profiles of components can be obtained separately by measurements or computed based on structural models, their volume fractions and structural models can be obtained. Conformational polydispersity can be described in terms of *R*_g_ and *D*_max_ distributions and using a set of representative structures.

In contrast to many other structural methods, SAXS is able to build meaningful models based on data collected from polydisperse samples, including variability in conformations and in species. The overall parameters and the scattering profile of a polydisperse system reflect a weighted average over the molecular ensembles of different types (Fig. 3 ▸). Knowledge about the type of polydispersity is required for an adequate interpretation of the SAXS data.

The scattering intensity *I*(*s*) of a conformational or oligomeric mixture is a linear combination of individual contributions *I_i_*(*s*) from the species, 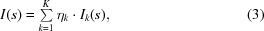
where η_*k*_ is the volume fraction of species *k* and *K* is the total number of components (Svergun *et al.*, 2013 ▸). In many practical cases (such as equilibrium oligomeric mixtures), *K* is reasonably small and information about the components, for example the dissociation products of a complex or the intermediates in a dynamic process, is available. If the structures of the components are known or their individual scattering profiles can be measured, the volume fractions of the species that fit the SAXS data can be found by solving (3). This approach has been implemented, for example, in the program *OLIGOMER* (Konarev *et al.*, 2003 ▸), utilizing nonnegative least squares to obtain the volume fractions, which in turn can be used to define the binding affinity (*K*_d_) values of protein-oligomerization processes. If the number of components *K* is not known *a priori*, methods such as singular value decomposition (SVD) or principal component analysis (PCA) may help to estimate this number (Henry & Hofrichter, 1992 ▸; Jolliffe, 2002 ▸). Recently, several novel approaches have been proposed to decompose SAXS data if the scattering profiles of the components are not available (Blobel *et al.*, 2009 ▸; Malaby *et al.*, 2015 ▸; Herranz-Trillo *et al.*, 2017 ▸). Notably, one of the latest major developments in SAXS studies of oligomeric systems is the possibility to structurally model the quaternary structures of components from equilibrium mixtures of partially dissociated complexes (Petoukhov & Svergun, 2013 ▸). Here, the experimental profile (or a series of profiles taken at different solute concentrations) is fitted by a weighted sum of scattering intensity contributions (3) from the assembled complex together with those from the individual components. Modelling can be performed *ab initio* using DRs to represent monomers in a symmetric homo-complex (*GASBOR_MX*) or with rigid-body refinement for hetero-complexes (*SASREF_MX*). In addition to the positions and orientations of the components in a complex, the volume fractions of the molecular species are determined.

In the structural analysis of flexible systems, *K* may reach astronomical numbers (for example for unfolded proteins) and the exact conformations are not known. For such systems, an approach has been proposed (Bernadó *et al.*, 2007 ▸) in which a large random pool of possible conformations of the target molecule are generated to represent the available search space. A set of structures whose combined scattering optimally fits the data is then selected using a heuristic search line genetic algorithm. This principle is utilized in the original ensemble-optimization method (*EOM*; Bernadó *et al.*, 2007 ▸), and in different subsequent implementations (Pelikan *et al.*, 2009 ▸; Yang *et al.*, 2010 ▸; Różycki *et al.*, 2011 ▸) with varying pool-generation and ensemble-selection procedures. The generated pool has to cover the possible conformation space containing structures that are physically and biologically feasible (atomistic models without steric clashes); if available, high-resolution models (for example of individual domains) can be included. The latest version of *EOM* (Tria *et al.*, 2015 ▸) contains an artificial intelligence-based approach for the generation of missing flexible regions without any size limitations either as random or native-like chains. The new *EOM* also provides useful quantitative measures of the flexibility of the selected ensemble compared with the pool.

In addition to the computational developments, an experimental approach using in-line size-exclusion chromatography (SEC), originally described by Mathew *et al.* (2004 ▸) and David & Pérez (2009 ▸), has further boosted the use of SAXS to study mixtures and equilibrium systems. Here, SAXS profiles are collected continuously while eluting a sample from a column and flowing it through the measurement cell. The SEC–SAXS setup that separates the signals from different components in a mixture is now offered at several bioSAXS-oriented SR beamlines. Significant efforts have been put into developing data-collection and analysis strategies to manage and analyze the thousands of SAXS data frames acquired over an elution profile (David & Pérez, 2009 ▸; Jensen *et al.*, 2010 ▸; Round *et al.*, 2015 ▸; Graewert *et al.*, 2015 ▸; Malaby *et al.*, 2015 ▸). The SEC–SAXS setup at P12 (PETRA III/EMBL/DESY) is equipped with a triple-detector array (TDA) to combine data from ultraviolet–visible light (UV–Vis) spectroscopy, refractive-index (RI) and right-angle light-scattering (RALS) detectors to determine the concentration and molecular weight (MW) of the eluted samples (Graewert *et al.*, 2015 ▸). The *R*_g_ and forward scattering *I*(0) are automatically extracted for each frame and correlated with the TDA results, yielding a so-called SEC–SAXS chromatogram. The MW estimates over the SEC–SAXS chromatogram peaks allow accurate determination of the oligomeric states of the component being eluted. Overlapping peaks containing scattering contribution from two or more species may cause complications in the analysis. To decompose the contributions from different overlapping components in a SEC–SAXS chromatogram several techniques have been applied, from Gaussian fitting of peaks to SVD and evolving factor analysis (Brookes *et al.*, 2016 ▸; Malaby *et al.*, 2015 ▸; Meisburger *et al.*, 2016 ▸).

## 3. Time-resolved SAXS: pushing the limits of temporal resolution   

Time-resolved SAXS (TR-SAXS) is the application of SAXS which has profited most from the recent developments in synchrotron instrumentation. TR-SAXS is an ideal tool to address the extremely important question of motion, which plays the role of a link between structure and function in biological molecules. The technique offers the possibility of analysis under quasi-physiological conditions, (sub)nanometre spatial resolution and an extremely broad range of time resolution from femtoseconds to days (Spilotros & Svergun, 2016 ▸). The main aspects considered in the design of TR-SAXS experiments include (i) the need to collect interpretable signals in short times and (ii) the need for a rapid trigger to initiate the reaction of interest over a statistical ensemble of molecules. Third-generation synchrotrons are able to provide X-ray pulses of as short as 100 ps and a flux of up to 10^15^ photons s^−1^ by using multilayer monochromators (MLMs; Blanchet *et al.*, 2015 ▸). Together with fast detectors, these features offer the possibility of very efficient data collection. Figs. 1 ▸(*b*) and 1 ▸(*c*) illustrate the time-resolved possibilities of modern SR by showing the SAXS profile of lysozyme (at 6.08 mg ml^−1^ concentration) collected with an MLM on the P12 beamline at PETRA III, which provides a flux at the sample position of ∼4 × 10^14^ photons s^−1^ at 10 keV. The profile in Fig. 1 ▸ was taken on an EIGER 4M pixel detector (frame rate 750 Hz, such that the exposure time was only 1.3 ms). Remarkably, the quality of the data set obtained as a standard ‘single shot’ is sufficient to perform a complete SAXS analysis of the low-resolution structure of the protein.

A key element in TR-SAXS experiments is a triggering mechanism to start and synchronize dynamic processes. The pump-and-probe method (in which an excitation signal is followed by a single probing pulse or a train of pulses) takes further advantage of the high brilliance of third-generation synchrotrons. Short and intense X-ray pulses are isolated by mechanical choppers or fast-gated detectors (Cammarata *et al.*, 2009 ▸; Westenhoff *et al.*, 2010 ▸). Fourth-generation sources such as X-ray free-electron lasers (XFELs) are able to produce extremely short (10 fs) X-ray pulses with a number of photons that is 10^2^–10^3^ times higher than the pulses typically produced at synchrotrons and a beam size as low as 0.1 mm. Sequential exposures can also investigate much slower dynamical processes on timescales longer than a second, which may easily extend to hours/days. Examples in the literature include amyloid fibrillation of various proteins such as insulin and α-synuclein (Vestergaard *et al.*, 2007 ▸; Herranz-Trillo *et al.*, 2017 ▸).

Rapid changes in temperature or pressure trigger a plethora of biological reactions. Temperature jumps can be achieved by mixing solutions at different temperatures (Δ*T* = ±40°C; dead time in the millisecond range), by infrared laser-induced heating or by employing inert absorbing dyes with fast internal energy conversion (Kubelka *et al.*, 2009 ▸). Pressure induces phase transitions in many condensed-matter systems: examples in the field of the phase behaviour of protein solutions, folding and configurational energy landscapes have been reviewed (Meersman *et al.*, 2006 ▸). However, pressure jumps using biological samples are technically challenging and are less commonly employed in kinetic studies. Ice formation, which occurs at around 1–1.5 GPa, limits the maximum hydrostatic pressure applicable to biological solutions. Recent technical developments have enhanced the time resolution achievable to submillisecond (Möller *et al.*, 2016 ▸).

Rapid mixing experiments enable fast perturbations using relatively simple technology; mixing is an ideal method to change solvent conditions such as pH, salt or ion content and to induce protein–ligand or protein–protein interactions. Devices employing continuous-flow mixing (turbulent or laminar) inside micro-fabricated channels achieve a time resolution of around 100 µs and low sample consumption (Akiyama *et al.*, 2002 ▸). Different time points of the reaction correspond to different distances from the mixing point. Several examples of applications, especially to protein folding, have been reported (Nobrega *et al.*, 2014 ▸; Kathuria *et al.*, 2013 ▸).

Light-triggered experiments allow one to push the time resolution of TR-SAXS further (Cammarata *et al.*, 2008 ▸). The difference in absorption between X-rays and visible or UV light has to be taken into account to ensure uniform excitation in the volume probed. Thin samples are usually required and liquid jets represent a possible technical solution for both synchrotron and free-electron laser sources (Trebbin *et al.*, 2014 ▸). Fast laser pulses (with a duration of picoseconds to femtoseconds) are used to initiate structural changes and intramolecular reactions; the structural changes are typically manifested in the resolution range 3–0.5 nm, *i.e.* 2 < *s* < 15 nm^−1^ (the effect of solvent heating usually limits the range of usable data). The potential of this approach has been shown in the study of the quaternary transition in carbonmonoxy haemoglobin (Cammarata *et al.*, 2008 ▸).

Other examples include similar experiments on carbonmonoxy myoglobin and the study of the photoactive yellow protein (PYP) photocycle (Cho *et al.*, 2010 ▸, 2016 ▸). A more recent study on signal amplification and transduction in phytochrome photosensors (Takala *et al.*, 2014 ▸) highlights how the combination of TR-SAXS and time-resolved crystallo­graphy with molecular dynamics (MD) simulations can be applied to characterize large-amplitude protein motions.

TR-SAXS data analysis requires specifically tailored modelling approaches. On timescales longer than picoseconds, TR-SAXS probes the exchange in population between finite numbers of local minima in the free-energy landscape. To isolate the pattern of a single species, one can make use of SVD or a global analysis based on a specific kinetic model for the reaction considered (Levantino *et al.*, 2012 ▸). On ultrafast timescales (femtoseconds to picoseconds) each time point corresponds to a well defined energy minimum. Once the profile of the single species has been isolated, it is possible to use different approaches. TR-SAXS data can be validated and interpreted by classical molecular-dynamics simulations (MD) or restrained non-equilibrium MD. Recent developments such as speeding up the calculation of theoretical X-ray scattering profiles by an accurate coarse-grained representation are improving the integration of SAXS data in MD simulation by reducing the computational complexity (Niebling *et al.*, 2014 ▸). Based on MD simulations, recent data on the protein quake after carbon monoxide photodissociation in myoglobin have been reinterpreted as a single pressure peak that propagates anisotropically (on a subpicosecond timescale) across the protein and further into the solvent (Brinkmann & Hub, 2016 ▸).

## 4. Validation and archiving   

Given the rapidly growing applications of SAXS in structural biology, it is of great importance to have common criteria for the integrity of SAXS data and models. Quality criteria are required to be able to estimate how detailed biological interpretation can be performed based on a SAXS model and how it can be further utilized in combination with other methods. In addition, taking special care of data standards and quality indicators is extremely important for archiving SAXS-based models and making them available to the community. Here, we go through the different aspects of SAXS data and model validation in more detail.

### 4.1. Data validation   

The starting point for SAXS analysis and modelling is the check of sample and data quality. The purity and ideality of a sample can be assessed using biophysical and analytical methods such as MALS, SDS–PAGE and DLS (Jeffries *et al.*, 2016 ▸) and also from the data themselves. Linearity of the Guinier plot in the range *s* < 1/*R*_g_ must be observed for a monodisperse sample (although linearity on its own does not guarantee monodispersity) and the independence of *R*_g_ values of concentration suggests absence of interparticle inter­actions. Deviations from the Guinier plot linearity are usually signs of aggregation (an upward turn) or repulsive interactions (a downward turn). Also, comparison of the MW estimates based on independent parameters, for example the *I*(0) value and the excluded volume *V*_p_ of a particle, with the theoretically expected MW provide a useful sanity check. The calculated *p*(*r*) function yields further indications of data quality, and aggregation is often detected through problems in the behaviour of *p*(*r*) at long distances. The above procedures are essential prerequisites for further analysis in terms of three-dimensional models, and if the necessary conditions are not fulfilled one must extrapolate the SAXS data to zero concentration using relevant procedures or, in the worst case, repurify the samples.

The SAXS data range to be measured is related to the dimensions of a particle and to the chosen modelling technique. A scattering profile must be measured at angles smaller than *s* = π/*D*_max_ to capture the size of a particle. On the other hand, the maximum *s* value (*s*_max_) required depends on the expected level of model detail. The needed data range for SAXS-based *ab initio* modelling is typically *s*_max_ = 8/*R*_g_ for dummy-bead models, while *s*_max_ up to 0.5 Å^−1^ is used for dummy-residue models. Rigid-body modelling employing high-resolution structures utilize nominal resolutions to about 10–20 Å, *i.e.* *s*_max_ values of 0.3–0.6 Å^−1^. In general, increasing the data range means increasing the information content and an improvement in the model detail. When studying secondary-structure elements and the changes in their arrangements, WAXS profiles are collected at higher angles (beyond *s* = 1 Å^−1^; note that WAXS measurements typically require elevated sample concentrations). In practice, the useful angular range is often limited by the SNR of the data, which critically depends on the experimental conditions (solute concentration, background *etc*). Recently, a method has been developed to determine the useful data range for an experimental data set by employing the Shannon sampling theorem (Konarev & Svergun, 2015 ▸).

### 4.2. Quality of SAXS-based structural models   

When reconstructing three-dimensional models from experimental SAXS data, one should never forget that modelling based on one-dimensional data is inherently ambiguous. A quantitative measure of the ambiguity associated with a scattering profile has recently been proposed based on a library of scattering profiles from shape skeletons describing a large quantity of low-resolution molecule-shape topologies (Petoukhov & Svergun, 2015 ▸). The potential ambiguity is measured as the number of the topologies compatible with the given experimental data. As a rule of thumb, highly oblate structures appear to be the most difficult for *ab initio* reconstruction from SAXS data.

Additional restraints and constraints based on available information (for example particle symmetry and/or anisometry) help to limit the search space, to reduce ambiguity and to speed up the calculations in *ab initio* modelling. Caution should, however, be taken when using constraints because incorrect restrictions can produce incorrect models that would still fit the data equally well. Typically, *ab initio* modelling is performed several times using different random seeds to produce and analyse variations in the restored models. The post-processing of multiple reconstructions is performed by their superposition using a normalized spatial discrepancy NSD (Kozin & Svergun, 2001 ▸) as a measure of dissimilarity between individual models and for classifying models into distinct structural clusters and subsequent averaging (Volkov & Svergun, 2003 ▸; Petoukhov *et al.*, 2012 ▸).

The clustering and averaging procedures assess the variability of *ab initio* models but not their resolution. In X-ray crystallography, the resolution is defined based on the highest diffraction peak that can be distinguished from the background and fitted by the crystallographic model. It is clear that this definition does not work for SAXS, and the 2π/*s*_max_ value is only a nominal theoretical limit of resolution that can never be achieved. Recently, a Fourier shell correlation (FSC) method that is used to assess the resolution of cryo-EM-based models was successfully extended to *ab initio* SAXS modelling (Tuukkanen *et al.*, 2016 ▸). The FSC of two models in real space is a measure of the correlation of their Fourier images in reciprocal space. In general, the FSC decreases with increasing momentum transfer, reflecting the loss of structural similarity with increasing resolution. The average FSC function over an ensemble of *ab initio* models reflects their variability. This has been demonstrated to be directly related to the resolution of the individual models in a model ensemble by extensive model calculations (Tuukkanen *et al.*, 2016 ▸). The program *SASRES* evaluating the resolution for bead and dummy-residue *ab initio* model systems is presently included in the above-mentioned averaging procedures and the resolution values are recommended to be reported in publications and depositions of SAXS data and models.

In a similar manner to *ab initio* modelling approaches, rigid-body modelling should be repeated multiple times and followed by structural clustering, providing an estimate of stability and revealing common model features. However, the frequent occurrence of a certain conformation does not necessarily indicate that it is the most native-like model. The reliability of rigid-body modelling improves significantly when it is applied in combination with additional information, especially with experimental and/or computational data on the interdomain or intersubunit contacts. An example of the use of a hybrid approach to validate and further analyse rigid-body models on an atomic level is the SAXS study of the interaction between transglutaminase 2 (TG2) and an anti-TG2 antibody derived from a single gut IgA plasma cell of a coeliac disease patient (Chen *et al.*, 2015 ▸). The program *SASREF* was used to reconstruct TG2–antibody complex models using the crystal structures of the antibody Fab fragment and TG2–GTP as a starting point. The models generated without any distance constraints could be classified into six different groups based on their binding interfaces. Previous biological knowledge of the Fab-fragment interaction-sequence motif indicated that four of the models classified into a single group represent the most native-like binding orientation. The predictions of interacting amino-acid residues based on the SAXS structures were further validated with results from hydrogen/deuterium-exchange experiments, mutagenesis studies and MD simulations. SAXS results can also be further improved by scoring rigid-body model ensembles in a post-processing step with the help of computational approaches (Oliva *et al.*, 2013 ▸; Vajda & Kozakov, 2009 ▸).

Making published SAXS data and models freely available to the scientific community is a central task (Trewhella *et al.*, 2013 ▸). The first database developed for SAXS models, BIOISIS (Hura *et al.*, 2009 ▸; http://www.bioisis.net), contains about 100 entries available for viewing and download. Presently available for archiving and retrieval is the Small-Angle Scattering DataBase (SASBDB; http://www.sasbdb.org; Valentini *et al.*, 2015 ▸), which contains over 350 entries depicting over 600 models (with over 150 entries on hold). For each entry in SASBDB, an experimental data file is stored together with the associated model-free parameters and possible *ab initio*, rigid-body or ensemble models, in accordance with the recommended guidelines for the information to be reported in SAXS publications (Jacques *et al.*, 2012 ▸). Further harmonization of standards in SAXS and integration with other structural biology methods is expected within the framework of the Hybrid/Integrative Methods Task Force of the wwPDB (Sali *et al.*, 2015 ▸).

## 5. Conclusions and future outlook   

The interest of the scientific community in the use of biological solution SAXS has steadily increased during the last decade. The popularity of the method has been propelled by the advanced experimental facilities (largely dedicated bioSAXS stations on brilliant SR sources) and also by novel data-analysis and modelling algorithms. Many of the programs that have been mentioned in this review are publicly available to academic users through the *ATSAS* package (https://www.embl-hamburg.de/biosaxs/software.html; Franke *et al.*, 2017 ▸).

Standard SAXS experiments and data analysis of monodisperse ideal samples can be fully automated at modern beamlines, moving the focus of SAXS research to tackle more difficult structural targets. Very importantly, SAXS can be utilized to obtain quantitative structural information on complicated systems. For flexible, oligomeric, interacting or evolving objects, SAXS often yields unique structural insights, and this makes the method an indispensable complementary tool to other higher resolution techniques such as MX, NMR or cryo-EM. In addition, the advent of XFELs has opened new possibilities for TR-SAXS to reach femtosecond time resolution (Neutze, 2014 ▸). The first studies have revealed conformational changes in proteins on the subpicosecond timescale using light-activated reactions (Arnlund *et al.*, 2014 ▸; Levantino *et al.*, 2015 ▸). Another promising technique for exploring evolving multi-phase systems is anomalous SAXS (ASAXS), which has become available owing to improved possibilities for tuning energies at modern synchrotron sources. In general, the synergistic use of complementary structural information provides information about biological systems over broad size ranges as well as spatial and temporal resolutions.

Modern SAXS is a powerful tool for structural systems biology in helping us to understand biochemical processes on the structural level in complex networks of interactions. Archiving SAXS/SANS data and models in publicly available databases will facilitate the use of SAXS for large-scale studies. This is also a major movement in the community towards broader use of the method in combination with complementary techniques and enabling the cross-validation of structural data. In this respect, the assurance of model and data quality has become an increasingly important question in SAXS practice. These problems are being addressed by the structural biology community within a global effort on the use of hybrid methods for integrative modelling (Sali *et al.*, 2015 ▸).
